# Influence of relative strength on the optimal load of the hang power clean and hang high pull in collegiate athletes

**DOI:** 10.3389/fspor.2025.1597535

**Published:** 2025-09-05

**Authors:** Yongmin Xie, Xingyu Pan, Fan Peng, Qinchang Sun

**Affiliations:** School of Strength and Conditioning Training, Beijing Sport University, Beijing, China

**Keywords:** optimal load, relative strength, hang power clean, hang high pull, strength and conditional training

## Abstract

**Background:**

Although training load is a critical determinant of adequate training stimuli for athletes, the optimal load for power training varies across individuals, and the underlying factors contributing to this variability remain unclear.

**Objective:**

This study investigated the influence of relative strength on optimal load during the execution of the hang power clean (HPC) and hang high pull (HHP) among college athletes.

**Methods:**

A total of 30 male college athletes (mean ± standard deviation age, 21.8 ± 2.3 years) performed hang power cleans (HPCs) and hang high pulls (HHPs) on a three-dimensional force plate at loads corresponding to 45%, 65%, 80%, and 95% of their one-repetition maximum (1RM), presented in a randomized and counterbalanced order. The relationship between optimal load and relative strength was assessed using Pearson's correlation coefficient.

**Results:**

The optimal load for achieving maximum output power in the hang high pull (HPP) and hang power clean (HPC) exhibited a highly individualized characteristic.A significant positive correlation was found between athlete strength and optimal load for both the HPC (*r* = 0.478, *P* < .01) and HHP (*r* = 0.611, *P* < .001).

**Conclusions:**

A positive correlation between optimal load and the relative strength of the athlete for the HPC and the HHP indicated that as the athlete's strength increased, the load intensity should be appropriately increased to maintain efficient training stimulation to elicit maximum power for each athlete. However, this study did not examine female collegiate athletes and other athletic populations. coaches should be discreetly when applying this conclusion to these athletic groups.

## 1 Introduction

The assessments of training load and mechanical power production in strength training are important to scientists, athletes, and coaches (1). The load that elicits maximal power production in a specific movement is commonly called the optimal load (2). Optimal load training, also known as maximum power training, is a strength training method focused on the best combination of load and speed, and such training can substantially improve athletic performance in explosive sports (3–5). To understand how load affects training stimuli, many investigators have evaluated the effect of load on peak force, velocity, power, and the rate of development during weightlifting exercises (2, 6–8). Researchers have also examined how load independently influences peak power of the bar, body, and system (bar plus body) (6). Such studies have provided a scientific basis for load selection in weightlifting and its derivative exercises. However, although it is generally believed that optimal load will be affected by factors such as athlete strength and movement skills, there are many gaps in knowledge regarding the contributions of these factors (5).

In practice, choosing an effective training load according to the stages of the training periodization is the key to effective training. As the training periodization progresses, the relative strength of the athlete gradually changes. How to adjust the training load according to the change in the relative strength of the athlete has becomed an important training problem remained to be solved. Thus, the present study explored how relative strength affects optimal load in two weightlifting derivative exercises that are used by athletes to improve lower-body power, the hang power clean (HPC) and the hang high pull (HHP).

## 2 Materials and methods

This study was undertaken with the understanding and written consent of each participant and conforms to The Code of Ethics of the World Medical Association (Declaration of Helsinki), printed in the British Medical Journal (18 July 1964).

### 2.1 Participants

This study recruited male undergraduate students from Beijing Sport University who participated in basketball, volleyball, swimming, track and field, weightlifting, table tennis, boxing, or other sports. The inclusion criteria were as follows: participants (1) had at least 6 months of training experience in HPC and HHP and were willing to accept professional evaluation and guidance of their movement technique; (2) were experienced in the maximum strength tests for HPCs and squats; (3) were free of any disease, with no sports injury within the prior 3 months; and (4) understood the content and procedures of this study and volunteered to participate in this research. The study was followed the Declaration of Helsinki and was approved by the Ethics Review Committee of Beijing sport University (No. 2024332H). All participants had read the experimental instructions and informed consent was signed before the beginning of the experiment.

### 2.2 Experimental procedures

This study comprised five sessions: (1) preparation, (2) anthropometric measurements, (3) one repetition maximum (1RM) squat, (4) 1RM HPC, and (5) power testing. In order to maintain the accuracy of the data and to prevent injuries, all tests were instructed and supervised by the same researcher, who was experienced with weightlifting training and testing.

#### 2.2.1 Preparation session

All participants completed a preparation session to become familiar with the test devices and the standard techniques for the squat, HPC, and HHP. A researcher experienced in strength training was responsible for demonstrating and monitoring the standard exercise techniques. The formal experimental sessions began a few days after the preparation session.

#### 2.2.2 Anthropometric measurement session

The body height, load, and composition of each participant was assessed. The session took place in the Scientific Research Center of Beijing Sport University. The participants arrived fasted (no food or water overnight) at 8:00 am. The measurement for height was accurate to centimetres (cm); and for body weight, to grammes. The body weight and body composition index were determined using an InBody 720 body composition analyzer.

#### 2.2.3 Squat 1RM

Participants used the standard squat technique demonstrated during the preparation session. Before the start of the squat test, all participants underwent a standardized warm-up. The warm-up began with 5 min of jogging, followed by dynamic stretching, such as lunges, side lunges, and squats.

To ensure the safety of the participants, two assistants experienced in strength testing stood on either side of the barbell and provided assistance when necessary. The take-off weight was based on the assessment of the participant (9). The same was true for the extent of the increase or decrease after each test trial. A successful test trial was one in which the participant lifted the weight for one repetition with the standard technique described to them before the experiment. An unsuccessful test trial was one in which the participant could not lift the weight for one repetition or lifted the weight with poor technique. There was a 3 min break between each trial. Each participant underwent one trial, was tested separately, and was asked to achieve their maximal performance.

#### 2.2.4 HPC 1RM

**Figure 1:**
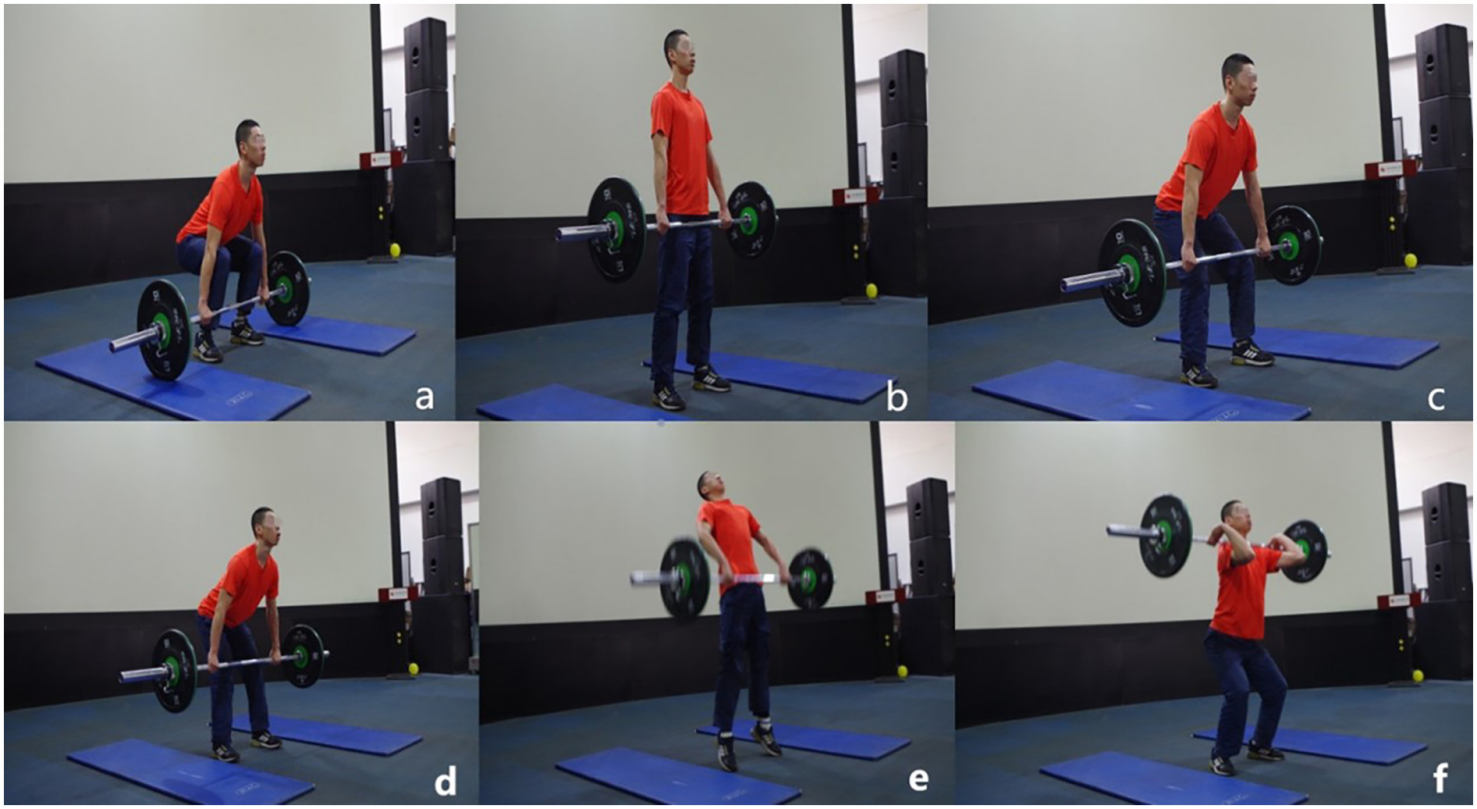
Illustration of the hang power clean. **(a)** Lifting the barbell from the floor. **(b)** Full stand with the barbell. **(c)** Lowering the barbell to the hang position. **(d)** Lifting the barbell with triple extension of the knee, hip, and ankle. **(e)** Full extension of the body. **(f)** Catching the barbell on the shoulders.

Participants used the standard HPC technique demonstrated during the preparation session (Figure 1). Participants should first stand with their feet shoulder-width apart and grip the bar with a clean grip using hook grip. Participants lifted the barbell from the floor to a standing position, then lowered the barbell to the just above the knee (hang position). After a pause of approximately 0.5 s, they lifted the barbell explosively in a vertical plane with extensions of the knee, hip, and ankle. Finally, they bent their knees and hips to “catch” the barbell on the shoulders in a quarter squat position (10). During the HPC 1RM test, a well experienced weightlifting coach stood besides the participants and give them instructions to make sure they perform correct technique. The HPC was termed unsuccessful if the researcher observed that the participant's upper thigh fell below parallel to the floor during the catch phase (11).

**Figure 2:**
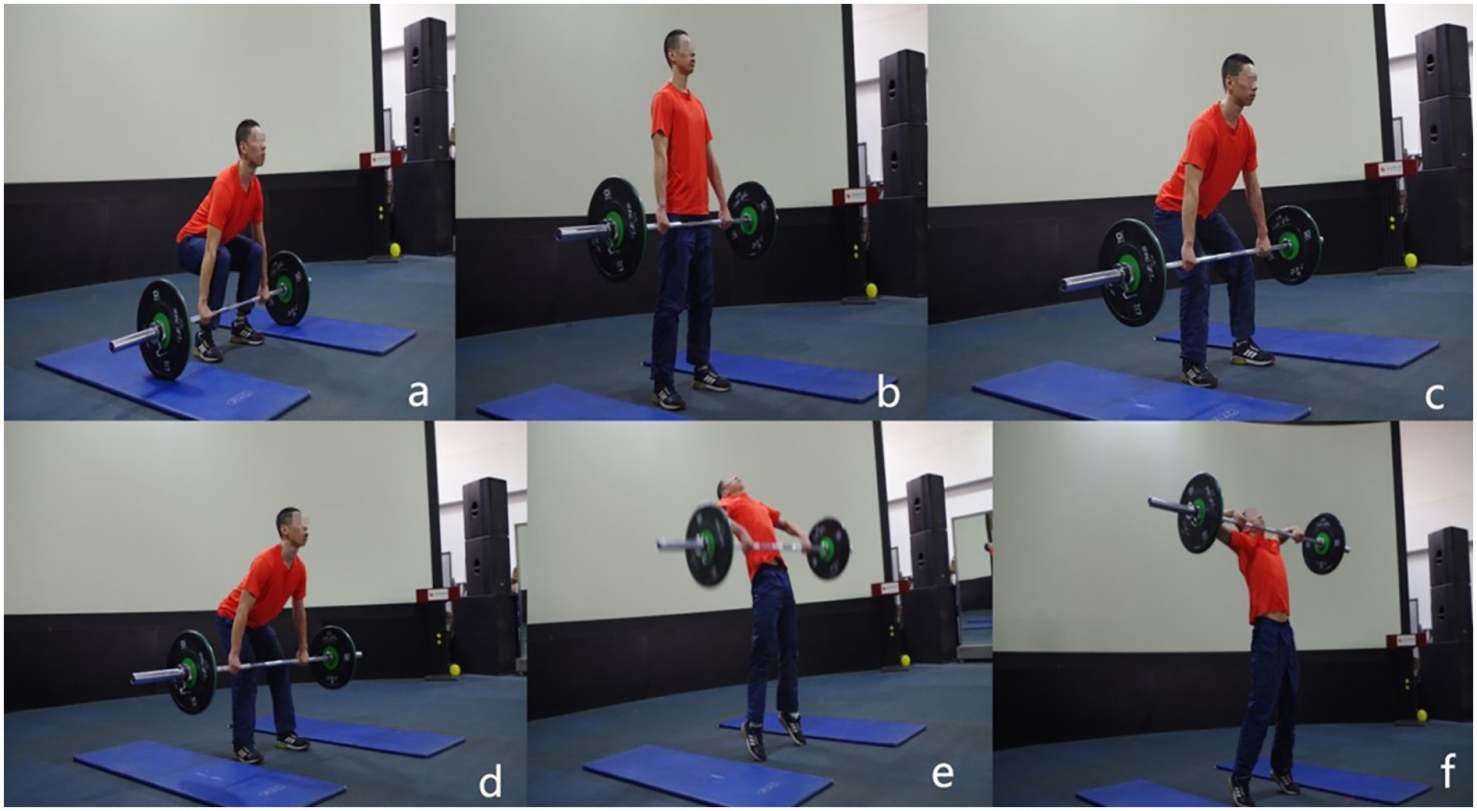
Illustration of the hang high pull. Panels **(a)** through **(e)** are the same as in Figure 1 Only panel **(f)**, which depicts the catch phase, differs. **(f)** Lifting the barbell to the highest point.

The HHP is a derivative exercise of the HPC. The two weightlifting exercises are similar except for the barbell catch phase (Figure 2). Because the HHP is a derivative exercise of the HPC, and it is difficult to judge the completion of the HHP repetition, this study used the HPC 1RM as the HHP 1RM. This is a common method for calculating the 1RM of HHP, and its effectiveness had been verified by relevant studies (7, 8, 12).

**Figure 3:**
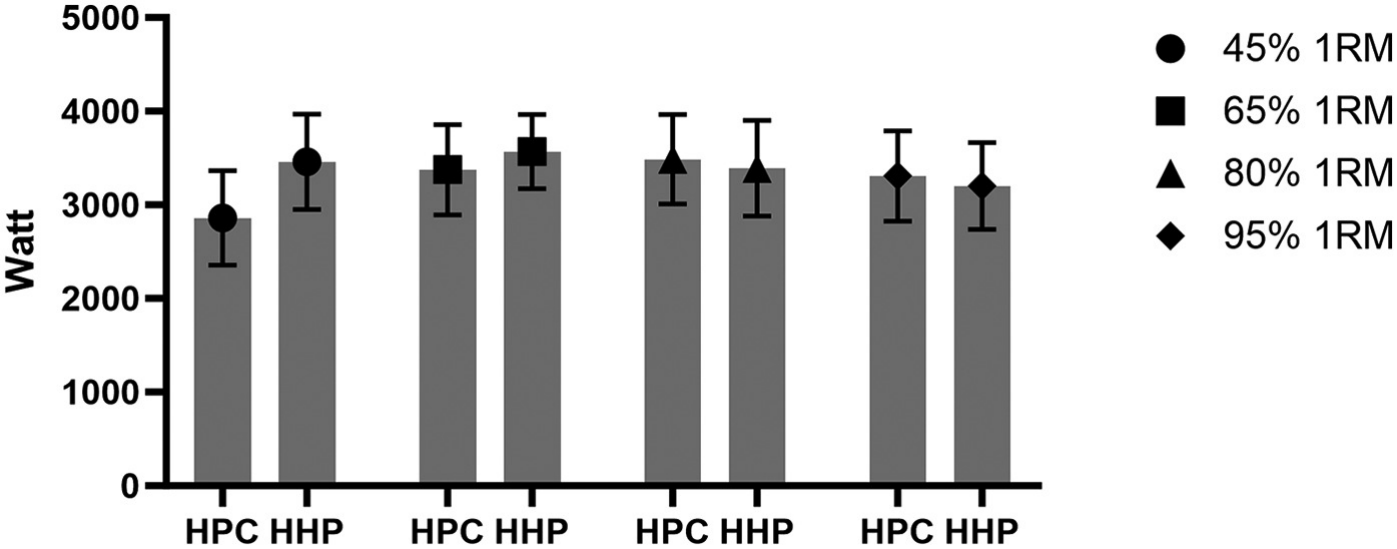
At increasing load intensity for the hang power clean (HPC) and hang high pull (HHP).

The test was carried out as follows: (1) participants performed a general warm-up for about 10 min, which included jogging and stretching and a low-intensity warm-up of 10 squats and 10 jumps; (2) participants warmed up with HPCs in the order of 30%, 40%, 70%, and 90% of the estimated maximum load of 1RM. The number of repetitions at each load intensity was 5, 3, 3, and 1, respectively; (3) Participants had four opportunities to obtain a 1RM and were allowed to rest for 3 min after each trial (13).

#### 2.2.5 Power testing session

Before the power test, participants completed the same warm-up as for the HPC 1RM test session. After a 10 min recovery period, they performed maximal-effort HPCs or HHPs on a three-dimensional force plate (Kistler 9281CA, 60 cm *90 cm*10 cm, Switzerland) of 45%, 65%, 80%, 95% of their predetermined 1RM in a random and balanced order which was operated by latin square method. The Kistler force plate had been used which was calibrated in a certified calibration laboratory less than 12 months ago. Force plate data were sampled at 1,000 Hz. The exercise techniques were the same as those described in the 1RM test session. A well experienced weightlifting coach would guard the teckniques for the whole test. Participants performed the test with two attempts at each load using a 3 min interval between attempts. Both test trails were recorded, and the one with the greatest power output was used for statistical analyses. In each trial, participants were encouraged to do their best. Owing to the large number of trials, only one exercise (HPC or HHP) was performed for each visit in a randomized order to prevent fatigue.

### 2.3 Data analysis

The numerical integration method was used to calculate the power output of the system (10). Numerical integration is the most common method for calculating the power output of weightlifting and its derivative exercises. With the vertical ground force during a certain interval and the mass of the system, the acceleration can be obtained. And then the speed change can be calculated. This change in velocity was then added to the center of gravity's previous velocity to produce a new velocity at time equal to that particular interval's end time (10).

Studies have shown that the accuracy of the initial integration speed and quality significantly affect the calculation results. In order to ensure the accuracy of the data, the participant must remain still for approximately 0.5 s before the second lift, and during this time, the mass of the entire system must be applied to the force plate (14).

When athletes perform exercises on the force plate, the force plate provides data on the ground reaction force corresponding to the time point. Through numerical integration of the ground reaction force over time, the power during the exercises can be calculated. This calculation method is often referred to as the forward dynamic method or the impulse-momentum method (15). In the numerical integration method, the ground reaction force exerted on the system (participant plus barbell) can be directly obtained through the three-dimensional force plate. Therefore, the mass of the athlete and the barbell must be on the force plate before the action starts.

The speed of the center of gravity of the system can be obtained through the integration of time. The most commonly used method is to calculate the change of the acceleration of the center of gravity of the system. The acceleration of the system (*a*) is equal to the ground reaction force (*F*) minus the gravity on the system (*W*) and then divided by the mass of the system, as shown in Equation (1)

(1) $$v=\int_{0}^{t}adt=\int_{0}^{t}\frac{\left(F-W\right)}{m}dt\;$$

For each time point (*i*), its power (*P*) is equal to the product of *F* and *V*, as shown in Equation (2).

(2) $$p_{\left(i\right)}=F_{\left(i\right)}\times V_{\left(i\right)}$$

Peak power refers to the maximum power output at any given point during the centripetal phase of the action. The centripetal process in this study was defined as the lowest point at which the barbell slid down to the highest point it was lifted. Because weightlifting and its derivatives focus on the maximum vertical movement distance of the system's center of gravity, this study required only the vertical ground reaction force to calculate the power of the system (10). The integration of the data began with the start of the action, and the integration constant of the initial speed was zero when the integration started. This is why the participants were required to pause for 0.5 s after they lowered the barbell to the hang position. Thus, in the calculation, the acceleration due to gravity is 9.8 m/s^2^. The load that elicited maximal power production in a HHP or HHC was defined the optimal load in the study.

### 2.4 Statistical analysis

All statistical analyses were conducted using SPSS version 20.0. Pearson's correlation tests were used to assess the correlation between relative strength and optimal load. A two-sided *P* < 0.05 was considered statistically significant.

## 3 Results

### 3.1 Participants

In total, 30 male undergraduate student athletes were included in the present study, including 3 national athletes, 5 national first-level athletes, and 22 national second-level athletes. Most of subjects were Sprinters. Others came from weightlifting, boxing, volleyball, basketball, table tennis, etc. The subjects had relatively well-proportioned figures, and there were no subjects with abnormal body shapes. Their mean (±standard deviation) age was 21.8 ± 2.3 years; height, 175.9 ± 7.2 cm; weight, 73.6 ± 5.1 kg; years of training, 5.2 ± 2.1; body mass index, 16.3 ± 2.5 kg/m^2^; maximum squat, 138.3 ± 33.3 kg; and maximum HPC, 87.0 ± 10.0 kg.

### 3.2 Relative strength

This paper used a relative strength index to evaluate the strength of each participant. Relative strength was calculated using the squat 1RM of each participant divided by their body weight. The mean (±standard deviation) of the weight of all 30 participants was 73.41 (±5.06) kg and of the squat 1RM was 140.17 (±35.66), for a calculated relative strength index mean (±standard deviation) of 1.91 (±0.47).

The 1RM back squat relative strength serving as one strength index had been widely used in Sports science research, and is significantly associated with speed-power measures and may be used as effective and practical indicators of athletic performance (16–18).

### 3.3 Effect of relative strength on the optimal load for HPC and HHP

The mean (±standard deviation) peak power output for the entire group of college athletes for the HPC and HHP as increasing load intensity (45% 1RM to 95% 1RM) is given in Table 1 and Figure 3.

**Table 1:** c at increasing load intensity for the hang power clean (HPC) and hang high pull (HHP) (*N* = 30).

Test	45% 1RM		65% 1RM		80% 1RM		95% 1RM	
	Mean	SD	Mean	SD	Mean	SD	Mean	SD
HPC (W)	2,860.62	504.98	3,375.68	481.44	3,486.01	477.15	3,309.29	481.11
HHP (W)	3,460.37	509.63	3,567.29	395.39	3,391.56	512.11	3,202.27	461.55

1RM, one repetition maximum; SD, standard deviation; W, watt.

The relative strength and optimal load for the HPC and HHP for all 30 college athletes combined are given in Table 2.

**Table 2:** Descriptive statistics for relative strength and optimal load of the hang power clean and hang high pull (*N* = 30).

Variable	Mean ± standard deviation	
	HPC	HPP
relative strength	1.91 ± 0.47	1.90 ± 0.47
Optimal load (% 1RM)	0.77 ± 0.11*	0.64 ± 0.15**

* Significant correlation between relative strength and optimal load for the HPC.

** Significant correlation between relative strength and optimal load for the HHP.

BW, body weight; RM, repetition maximum; HPC, Hang power clean; HHP, Hang high pull.

We performed Pearson's correlation test to assess the correlation between participant relative strength and optimal load for the HPC and HHP. The results indicated that there was a positive correlation between relative strength and optimal load for both the HPC (*r* = 0.478, *P* < .01) and the HHP (*r* = 0.611, *P* < .001).

## 4 Discussion

This study assessing how strength affects optimal load in two exercises commonly used by athletes and their coaches to improve lower-body power found power output levels for the HPC and HHP similar to that of previous studies (11, 13, 14). The power output of these two exercises could be up to more than 3,500 W, which indicated that these weightlifting derivatives may provide power training stimuli the same as Olympic weightlifting. The research also found that the HHP could elicit power as high as the HPC. This indicated that weightlifting pulling derivatives provide the same high power as weightlifting exercises even though the HHP exercises are easier to perform and to learn.

When examined the correlation between relative strength and optimal load for the HHP and HPC, the research found significant positive correlations for both exercises, with a correlation coefficient for the HPC of 0.478 and for the HHP of 0.611. These results indicated that the optimal load for college athletes varied with their increased or decreased relative strength. Thus, in practice, the training load intensity for the HPC and HHP should be changed based on the athlete's relative strength so as to always stimulate maximum power output.

The strong positive correlation found between relative strength and optimal load indicated that the greater the strength of the athlete, the higher intensity of the load is required to produce maximum power output. A study by Stone et al. also found that as relative strength increased, the load intensity corresponding to the maximum power output would also increase (19). Another study had shown that physically strong athletes may produce a maximum power output with a higher load intensity than relatively thin athletes (19).

As the relative strength of a college athlete increases, the load intensity which elicit the maximal power of the HPC or HHP should be increased accordingly. However, the selection of the optimal load is indeed a very complex task. Based on the trends of relative strength and the changes in the optimal load, this study suggests that novice athletes can start their training with 45% of their HPP or 65% of their HPC. As their relative strength and training level increase, they can gradually increase the optimal load to 65% of their HPP or 80% of their HPC, or even higher. It should be emphasized that coaches need to recognize the increase in load intensity, rather than merely focusing on the absolute load itself. With improvement in an athlete's abilities, simply increasing the load may not achieve the required increase in load intensity.

Another important finding of the research was the the large individual responses to the optimal load for maximum power output. This study did not conduct long-term observation on the subjects, so there was no sufficient evidence to illustrate how an individual's optimal load varies with relative strength. The current finding suggested that individual determination of athletes' optimal load was necessary to effectively develop their maximal power output.

## 5 Conclusion

Both HPC and HHP can generate high power output, indicating that they are both effective exercises for explosive strength training. However, the optimal load for HHP is approximately 65% of 1RM, which occurs at a lower intensity compared to the HPC's optimal load of around 80% 1RM.The optimal load of the HPC and HHP are significantly and positively correlated with the relative strength of the athlete, indicating that as an athlete's relative strength increases, the load intensity should be appropriately increased to maintain efficient training stimulation. Individual determination of athletes' optimal load is also necessary to effectively develop their power generating capabilities.

## 6 Practical application

In practice, a relatively low load intensity can be used in the initial stage of strength training for collegiate male athletes. With improvement in the athlete's relative strength, the training load can be gradually increased so that the load intensity always enables production of maximal power for the athlete. For example novice athletes may begin their training with loads corresponding to 45% of their HPP or 65% of their HPC. As their relative strength and training experience develop, it is recommended to progressively increase the optimal load to approximately 65% 1RM for HPP or 80% 1RM for HPC, and potentially beyond.

However, this study did not examine female collegiate athletes and other athletic populations. Coaches should be discreetly when applying this conclusion to these athletic groups. Further studies could be focused on female collegiate athletes and other athletic populations to better understand the effect of relative strength on optimal load. Coaches should also examine the optimal load in different stages of periodization and put optimal load training in the appropriate part of the periodization according the demands of sports.

## Data Availability

The original contributions presented in the study are included in the article/Supplementary Material, further inquiries can be directed to the corresponding author.
